# Supplementary material for: Mindfulness-Based Cognitive Therapy for Stress Reduction in Family Carers of People Living with Dementia: A Systematic Review
Source: Int J Environ Res Public Health. 2022 Jan 5;19(1):614. doi: 10.3390/ijerph19010614 (PMC8744610; doi:10.3390/ijerph19010614)
Supplement: Supplementary file 1 [file ijerph-19-00614-s001.zip › ijerph-1452229-supplementary.pdf]

International Journal of
Environmental Research and Public Health

MDPI

Supplementary Table S1. MEDLINE search strategy (OVID interface).

1. Mindfulness/
2. mindfulness.ti,ab,kw.
3. (MBCT or mindfulness based cognitive therapy or MBI or mindfulness based intervention? or MBP or mindfulness based program* or mindful*).ti,ab,kw.
4. or/1-3
5. Caregivers/
6. ((famil* or spous* or relative? or relation or relations or partner? or husband? or wife or wives or significant other? or child* or sibling? or grandchild*) adj5 (care* or caring or caregiv* or care giv*)).ti,ab,kw.
7. 5 or 6
8. dementia/ or alzheimer disease/ or dementia, vascular/ or cadasil/ or dementia, multi-infarct/ or frontotemporal lobar degeneration/ or frontotemporal dementia/ or "pick"/ or huntington disease/ or lewy body disease/
9. dementia.ti,ab,kw.
10. alzheimer?.ti,ab,kw.
11. (cognit* or neurocognit* or dement* or cadasil or frontotemporal or fronto temporal or pick? or huntington? or lewy-body or Parkinson? or primary progressive aphasia or carasil or Creutzfeldt Jakob or CJD or sundown* or Korsakoff or binswanger? or kluver bucy or primary progressive nonfluent aphasia).ti,ab,kw.
12. or/8-11
13. 4 and 7 and 12
